# Synthesis of Porous Materials Using Magnesium Slag and Their Adsorption Performance for Lead Ions in Aqueous Solution

**DOI:** 10.3390/ma16227083

**Published:** 2023-11-08

**Authors:** Guangjun Lu, Jingang Han, Ying Chen, Hongjiao Xue, Ruifang Qiu, Xinxing Zhou, Zhibin Ma

**Affiliations:** State Environmental Protection Key Laboratory of Efficient Utilization Technology of Coal Waste Resources, Institute of Resources and Environmental Engineering, Shanxi University, Taiyuan 030006, China; lgj275@sxu.edu.cn (G.L.); hanjingang105@163.com (J.H.); 15917529446@163.com (Y.C.); x15102564482@163.com (H.X.); qiurf@sxu.edu.cn (R.Q.); zxx09432338@whut.edu.cn (X.Z.)

**Keywords:** magnesium slag, geopolymer, porous materials, Pb^2+^, adsorption

## Abstract

Magnesium slag-based porous materials (MSBPM) were successfully synthesized using alkali activation and foaming methods as an effective adsorbent for Pb^2+^ in solution. The effects of foaming agent type, foaming agent dosage, alkali dosage, and water glass modulus on the properties of the MSBPM were studied, and the micromorphology and porosity of the MSBPM were observed using microscopy. The influence of pH value, initial concentration, and adsorbent dosage on the Pb^2+^ adsorption was investigated. The results showed that a porous material (MSBPM-H_2_O_2_) with high compressive strength (8.46 MPa) and excellent Pb^2+^ adsorption capacity (396.11 mg·g^−1^) was obtained under the optimal conditions: a H_2_O_2_ dosage of 3%, an alkali dosage of 9%, a water glass modulus of 1.3, and a liquid–solid ratio of 0.5. Another porous material (MSBPM-Al) with a compressive strength of 5.27 MPa and the Pb^2+^ adsorption capacity of 424.89 mg·g^−1^ was obtained under the optimal conditions: an aluminum powder dosage of 1.5‰, an alkali dosage of 8%, a water glass modulus of 1.0, and a liquid–solid ratio of 0.5. When the pH of the aqueous solution is 6 and the initial Pb^2+^ concentrations are 200~500 mg·L^−1^, the MSBPM-H_2_O_2_ and MSBPM-Al can remove more than 99% of Pb^2+^ in the solution. The adsorption process of both materials followed the Langmuir isotherm model and pseudo-second-order kinetic model, indicating that the adsorption process was a single-molecule layer chemical adsorption.

## 1. Introduction

With the continuous advancement of industrialization, a large amount of industrial solid waste is generated. Owing to the excellent physical properties, magnesium metal is widely used in aerospace, transportation, and electronic communication fields [1]. However, the production of 1 t of magnesium metal usually discharges 8~10 t of magnesium slag. Approximately 8 million tons of magnesium slag were discharged every year in China [2]. Currently, the utilization pathways of magnesium slag include its use as a supplementary material in cement production for the production of high-performance cement, or the utilization of the silica in magnesium slag for the production of glass, ceramics, and other silicate materials. However, due to issues such as processing costs, most of the magnesium slag has not been effectively treated, and the unutilized magnesium slag is stored and buried, leading to a waste of land resources and causing serious damage to the ecological environment [3]. Therefore, it is urgent to find an effective method to properly dispose of magnesium slag.

Metal lead (Pb) and its compounds are mainly used as raw materials in core industries such as battery manufacturing, metallurgy, and explosives manufacturing. While lead brings advanced products to humans, it also causes serious pollution to the environment. The intensification of industrialization and globalization has led to the release of heavy metals, especially Pb^2+^, into water bodies through various industrial processes, which is a worrying threat that can enter the human body and pose a risk to human health [4]. Excessive Pb^2+^ in the body can cause symptoms such as headaches and diarrhea, and irreversible damage to the liver, kidneys, and nervous system [5]. Therefore, it is necessary to remove this element from wastewater to prevent it from entering the environment. The concentration of lead ions in industrial wastewater varies by industry and process. The concentration of lead ions in wastewater produced by battery manufacturing, printing and dyeing industries, and electroplating industries is typically between 1 to 50 mg·L^−1^, 1 to 25 mg·L^−1^, and 8 to 10mg·L^−1^, respectively [6]. Research has been conducted in recent years to study the treatment of low-concentration lead-containing wastewater. However, in actual production processes, the concentration of lead ions in some electroplating industrial wastewater can reach several hundred milligrams per liter (mg·L^−1^). This concentration is influenced by various factors, such as different electroplating processes and the efficiency of wastewater treatment. A similar situation can occur in metallurgical industrial wastewater. It is important to note that the morphology and valence state of lead in the water body can also change due to environmental factors, which may lead to its re-release into the water body. Therefore, it is necessary to treat wastewater containing medium to high concentrations of lead [7]. Currently, there are various treatment methods available to remove Pb^2+^ from water, such as chemical precipitation [8], ion exchange [9], membrane separation [10], and adsorption [11]. Chemical precipitation can produce harmful sludge, causing secondary pollution. Ion exchange has the disadvantage of complexing agent contamination of ion exchange resins. Membrane separation is expensive and easily damaged. Adsorption is a widely available, economically effective, and green method for removing heavy metal ions from wastewater [12].

In recent years, the preparation of geopolymer functional materials using industrial solid wastes such as fly ash, coal gangue, and carbide slag by alkali activation has received widespread attention [13]. Industrial solid wastes exhibit certain cementitious properties after alkali activation, and its unique three-dimensional network structure and ion exchange properties make geopolymer applicable in environmental remediation fields such as adsorption, catalysis, and heavy metal solidification [14,15]. The common alkali activators are hydroxides (NaOH, KOH), silicates (Na_2_SiO_3_, K_2_SiO_3_), or a mixture of both [16]. Due to their porous structure and cation exchange with cations in solution, they can effectively adsorb heavy metal ions such as Pb^2+^ [17]. Currently, there is limited research on using magnesium slag to prepare geopolymer for adsorbing heavy metals in wastewater. Commonly used raw materials for adsorbents include fly ash, slag, etc. Xue [18] prepared slag-based geopolymer microspheres with particle sizes ranging from around 10 to 125 μm for Pb^2+^ adsorption. Their results showed that the adsorption capacity of the adsorbent for Pb^2+^ reached 353.9 mg·g^−1^, and the removal rate was 88.03%. Su [19] used sodium hydroxide and slag as raw materials to prepare geopolymer microsphere adsorbents, which had high adsorption capacities of 335.43 mg·g^−1^, 414.38 mg·g^−1^, and 91.21 mg·g^−1^, for Cu^2+^, Ni^2+^, and Co^2+^, respectively, in aqueous solution. The adsorption process conformed to the pseudo-second-order kinetic model and Langmuir isotherm model. Zhang et al. [20] studied the adsorption behaviors of cations at the N-A-S-H interface by molecular dynamics simulation. Results showed that electrostatic attraction and ion exchange were the main mechanisms for adsorption of metal cations. For cations with the same charge number, the ion radius was inversely proportional to cation exchange and adsorption capacity. Among cations with different charge numbers, cations with lower ion potential were more easily adsorbed on the gel surface. In summary, geopolymer has been proven to be an effective adsorbent, but its disadvantage is that the geopolymer structure is relatively dense, and the adsorption efficiency is low. Therefore, the preparation of porous geopolymer is proposed to improve the adsorption capacity of heavy metal ions. Currently, there is a relative lack of research on the preparation of porous adsorbent materials using magnesium slag. The understanding of the process and material properties of porous adsorbent materials prepared from magnesium slag is insufficient, including preparation methods, stability, durability, and adsorption mechanisms. Furthermore, the potential application fields of porous magnesium slag materials need to be further expanded to fully exploit their application potential.

This study focuses on the utilization of magnesium slag, a solid waste, as the primary raw material to prepare magnesium slag-based porous materials (MSBPM) through alkali activation and foaming using H_2_O_2_ and aluminum powder, respectively. The effects of foaming agent dosage, alkali dosage, and water glass modulus on the compressive strength, apparent density, and porosity of MSBPM were investigated. Furthermore, the adsorption performance of the adsorbents prepared by the two foaming methods for Pb^2+^ in aqueous solution was studied under different conditions.

## 2. Materials and Methods

### 2.1. Materials

The magnesium slag was obtained from the self-cooled slag of a magnesium refining plant in Fugu, Shaanxi Province, China. Chemical reagents contained sodium hydroxide powder (NaOH, MACKLIN reagent, GR, Shanghai, China), nitric acid (HNO_3_, Tianjin Damao Chemical Reagent Co., Ltd., GR, Tianjin, China), lead nitrate (Pb(NO_3_)_2_, Tianjin Comio Chemical Reagent Technology Co., Ltd., AR, Tianjin, China), water glass (Na_2_O·3.3SiO_2_, Shandong Yusuo Chemical Technology Co., Ltd., AR, Linyi, China), hydrogen peroxide (H_2_O_2_, Tianjin Obokai Chemical Co., Ltd., AR, Tianjin, China), and aluminum powder (Zhengzhou Yuhang Aluminum Co., Ltd., AR, Zhengzhou, China).

### 2.2. Synthesis of MSBPM

(1) Preparation of activator: A mixed activator of NaOH and Na_2_SiO_3_ was used, and the calculation method of the ratio has been given in the literature [21]. Water glass and deionized water were stirred in a beaker. An amount of 9.4~12.2 g of sodium hydroxide was slowly added to the mixed solution of water glass and deionized water. The mixed solution was stirred with a glass rod until it was fully mixed, and then was aged for 24 h before use.
(2) Preparation of MSBPM: 150 g of magnesium slag was added to the prepared activator, and then stirred at a speed of 1500 r·min^−1^ for 5 min in a high-speed disperser to obtain a uniformly mixed slurry. Then, 0.075~12 g of foaming agent was added, and the homogeneous slurry was quickly poured into a silicone mold with dimensions of 20 mm × 20 mm × 20 mm. The mold was then placed on a cement mortar vibrator for 30 s, covered with plastic film to prevent moisture loss, and finally placed in a constant temperature of 60 °C and humidity curing box for 24 h. After curing, the mold was removed to obtain a standard test block, which was placed in a constant temperature of 25 °C and humidity curing box for further curing until the preset time for subsequent testing.

### 2.3. Material Characterization

X-ray fluorescence (XRF, S8 Tiger, Bruker, Ettlingen, Germany) was employed to measure the primary chemical compositions of materials. Powder X-ray diffraction (XRD, D2, Bruker, Ettlingen, Germany) with Cu K_α_ radiation was used to identify the minerals in the specimens, which were scanned with a 2*θ* step size of 0.02° in the range of 10–80°. The morphology and regional composition of the sample were analyzed using a stereo microscope (Zeiss Stemi 2000C, Carl Zeiss, Jena, Germany). The concentration of Pb^2+^ before and after adsorption was determined by inductively coupled plasma emission spectrometry (ICP-OES, Icap6000, Thermo Fisher Scientific, Waltham, MA, USA). The compressive strength of MSBPM was tested using an automatic cement flexural and compressive testing machine (DYE-300S, Wuxi Huaxi Building Materials Test Instrument Co., Ltd., Wuxi, China).

### 2.4. Pb^2+^ Adsorption Experiments

The synthesized MSBPM exhibited weak alkalinity. To prevent the precipitation of Pb^2+^ from affecting the experimental results, the MSBPM was crushed and ground to below 100 mesh prior to the experiment. MSBPM powder was washed with deionized water in a beaker until neutral. The sample was then filtered and dried at 60 °C before being stored in a self-sealing bag for later use.

A volume of 50 mL of Pb^2+^ containing solution with a specific concentration and a certain amount of MSBPM were added to a 100 mL blue bottle. The detailed conditions of the adsorption experiment were listed in Table 1. The blue bottle was placed in a constant temperature water bath shaker at 25 °C and kept for 120 min at a shaking frequency of 200 r·min^−1^. The mixture was then poured into a 50 mL centrifuge tube and centrifuged at a speed of 7500 r·min^−1^ for 3 min. The supernatant was diluted 100 times after passing through a 0.45-μm filter membrane. The concentration of Pb^2+^ in the solution before and after adsorption was determined by ICP-OES, and all experiments were conducted three times.

The equilibrium adsorption capacity (*q*_e_, mg·g^−1^) and adsorption efficiency (*ƞ*, %) were calculated by the Equations (1) and (2), respectively:

(1) $$q_{e}=\left(C_{0}-C_{e}\right)\times \frac{V}{m}$$

(2) $$\eta =\frac{C_{0}-C_{e}}{C_{0}}\times 100\%$$

where *C*_0_ and *C*_e_ (mg·L^−1^) are the initial and equilibrium concentrations of Pb^2+^, respectively. *V* (L) is the initial volume of the aqueous solution, and *m* (g) is the adsorbent mass.

## 3. Results and Discussion

### 3.1. Properties of Raw Materials

**Figure 1:**
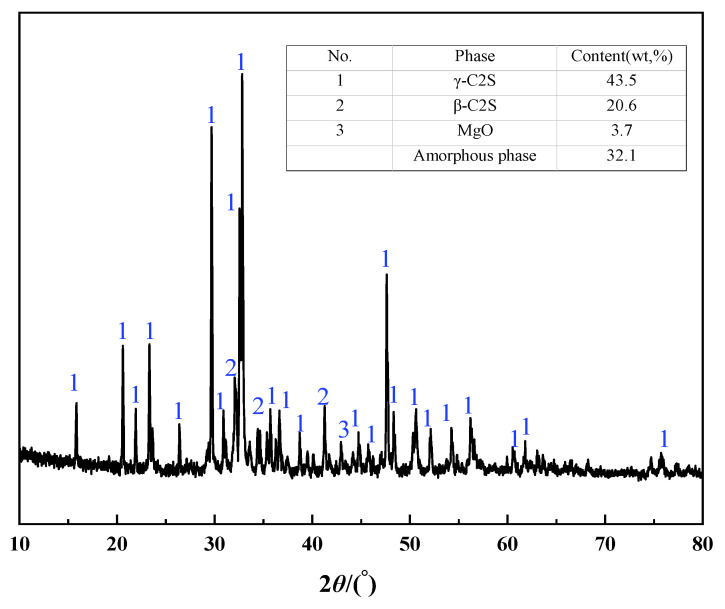
XRD pattern of magnesium slag.

Table 2 and Figure 1 showed the main chemical composition, mineral phase, and microstructure of magnesium slag. The content of CaO and SiO_2_ accounts for more than 90% of the total mass of magnesium slag. The crystal minerals in magnesium slag are mainly calcium silicate (2CaO·SiO_2_, 64.1%) and magnesium oxide (MgO, 3.7%). Among them, γ-C_2_S accounts for 43.5%, β-C_2_S accounts for 20.6%, and the remaining 32.1% exists in an amorphous phase. The amorphous phase component has poor stability, and its structure is easily destroyed, which is conducive to stimulating the activity of magnesium slag.

### 3.2. Synthesis of MSBPM

Based on previous experimental results, the water glass modulus of 1.0 and 1.3, alkali content of 8% and 9%, water-to-binder ratio (W/B = 0.5), and foaming agents of H_2_O_2_ and aluminum powder were selected for the preparation of MSBPM. Table 3 listed the detailed experimental parameters and 28 d compressive strength. The dosage of foaming agent is expressed as a percentage of the mass of magnesium slag, and the amount of additional water is calculated by subtracting the water content in the water glass from the total water demand. An MSBPM with a foaming agent dosage of 1%, an alkali dosage of 8%, and a modulus of 1.0 is represented as 1–8%–1.0.

#### 3.2.1. Effect of Hydrogen Peroxide Dosage, Alkali Dosage, and Modulus of Water Glass on the Performance of MSBPM

**Figure 2:**
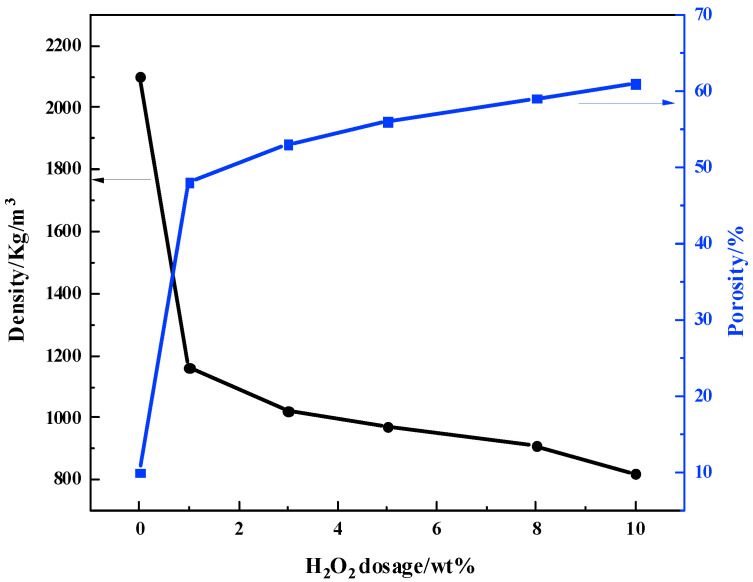
Variation curves of the apparent density and porosity of MSBPM with the addition of H_2_O_2_.

**Figure 3:**
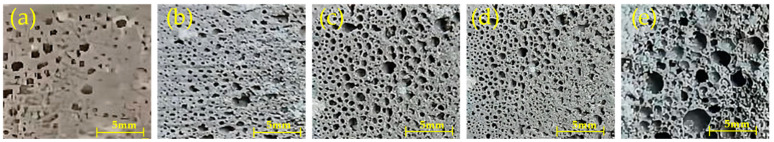
Micromorphology images of MSBPM under different H_2_O_2_ dosages: (**a**) 1%, (**b**) 3%, (**c**) 5%, (**d**) 8%, (**e**) 10%.

The decomposition of H_2_O_2_ at a certain temperature can generate foam and cause expansion of the material. The content of foaming agent has a significant influence on the compressive strength, apparent density, and porosity of the material [22]. As shown in Table 3, the compressive strength of MSBPM generally decreases with the increase in H_2_O_2_ dosage. When the combination of alkali dosage and modulus of water glass is 8%–1.0, 8%–1.3, and 9%–1.0, the addition of H_2_O_2_ leads to a significant decrease in compressive strength, which cannot meet the practical application requirements. When the alkali dosage and activator modulus is 9% and 1.3, respectively, the compressive strength is the highest at 8.46 MPa with a H_2_O_2_ dosage of 3%, and the compressive strength is the lowest at 1.80 MPa with a H_2_O_2_ dosage of 10%, which meets the requirements. Figure 2 showed the variations in the apparent density and porosity of MSBPM with an alkali dosage of 9% and an activator modulus of 1.3 under different H_2_O_2_ dosages. As the H_2_O_2_ dosage increases, the apparent density and porosity show a monotonic decrease and a monotonic increase, respectively, with corresponding ranges of 820~1160 kg/m^3^ and 61%~48%. Figure 3 showed the variations in the pore structure of MSBPM with an alkali dosage of 9% and an activator modulus of 1.3 after 28 d under the different dosages of foaming agent. As the H_2_O_2_ dosage increases, the number and size of pores increase, resulting in a loose and porous structure that is not conducive to the strength development. The pore size of MSBPM foamed by H_2_O_2_ is within the range of 0.1~2.4 mm.

#### 3.2.2. Effect of Aluminum Powder Dosage, Alkali Dosage, and Modulus of Water Glass on the Performance of MSBPM

Owing to the high content of alkali in alkali-activated binder system, aluminum powder is considered to be used to react with alkali to form hydrogen gas, which can form a large amount of pores [23].

**Figure 4:**
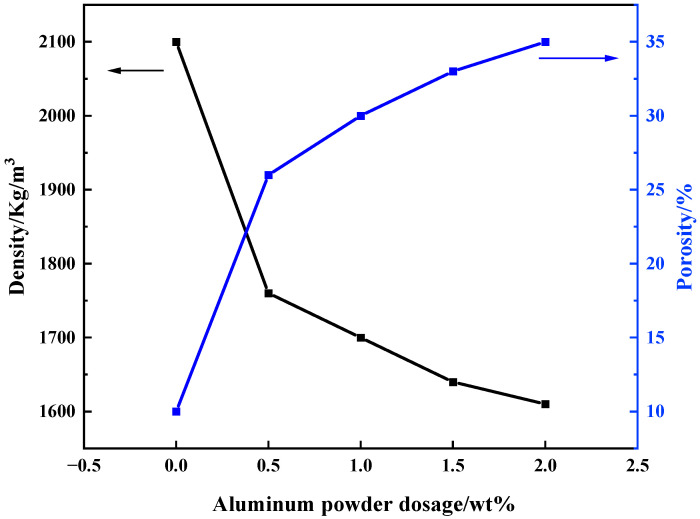
Variation curves of the apparent density and porosity of MSBPM with the addition of aluminum powder.

**Figure 5:**
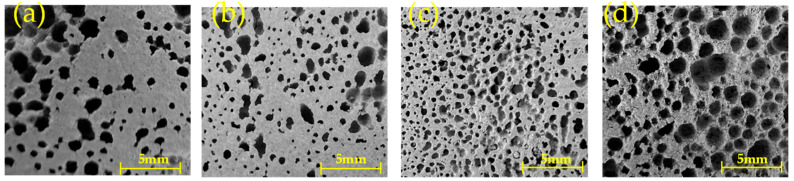
Micromorphology images of MSBPM under different aluminum powder dosages: (**a**) 0.5‰, (**b**) 1‰, (**c**) 1.5‰, (**d**) 2‰.

According to the Table 3, the compressive strength of MSBPM generally decreases with the increase in aluminum powder dosage. However, the addition of aluminum powder does not significantly reduce the compressive strength under the conditions of 9%–1.0 and 9%–1.3, indicating that the foaming agent does not play a foaming role. The solidification rate of the slurry is higher than the decomposition rate of aluminum powder, and, thus, the foaming of aluminum powder is limited to a small range within the network structure of the cementitious matrix [24]. The dynamic force of foaming is smaller than the resistance of the slurry, and the bubbles cannot escape or have already burst and collapsed within the slurry, resulting in incomplete foaming and no significant change in strength. The foaming effect of aluminum powder was obvious, and the specimens had a certain compressive strength when the alkali dosage and modulus of water glass are 8% and 1.3, respectively. And the highest compressive strength is 10.7 MPa when the dosage of aluminum powder is 0.5‰; the lowest compressive strength is 3 MPa when the dosage of aluminum powder is 2‰. Figure 4 showed the changes in apparent density and porosity of MSBPM with aluminum powder dosage under the condition of 8%–1.3. As the aluminum powder dosage increases, the apparent density and porosity show a decreasing and increasing trend, respectively, with corresponding ranges of 1610~1830 kg/m^3^ and 35%~23%. Figure 5 showed the changes in pore structure of MSBPM with foaming agent dosage after 28 d under the condition of 8%–1.3. As the aluminum powder dosage increases, the number and size of pores increase. The dosage of aluminum powder was negatively correlated with the compressive strength of the specimens. The pore size of MSBPM foamed by aluminum powder is within the range of 0.25~1 mm.

### 3.3. Adsorption Performance of the MSBPM

#### 3.3.1. Effects of Foaming Agent Dosage, Alkali Dosage, and Modulus of Water Glass

An MSBPM with certain strength and good pore structure from Section 3.2 was selected for adsorption experiments. The MSBPM was prepared under the alkali dosage of 9% and the activator modulus of 1.3 when H_2_O_2_ was used as the foaming agent, and under the alkali dosage of 8% and the activator modulus of 1.0 and 1.3 when aluminum powder was used as the foaming agent. The adsorption conditions were shown in Table 1, and the adsorption results were shown in Table 4. Considering both compressive strength and adsorption capacity, the suitable adsorbent material was selected. The adsorption capacity of the 3%–9%–1.3 sample was 396.11 mg·g^−1^, and the compressive strength was 8.46 MPa when H_2_O_2_ was used as the foaming agent. The adsorption capacity of the 1.5‰–8%–1.0 sample was 424.89 mg·g^−1^, and the compressive strength was 5.27 MPa when the aluminum powder was used as the foaming agent. The H_2_O_2_ porous material with a ratio of 3%–9%–1.3 was named MSBPM-H_2_O_2_, and the aluminum powder porous material with a ratio of 1.5‰–8%–1.0 was named MSBPM-Al for subsequent adsorption experiments.

#### 3.3.2. Effects of pH

**Figure 6:**
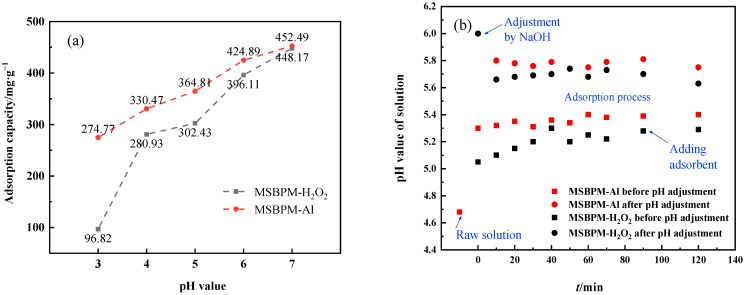
(**a**) Effect of pH on the adsorption capacity of Pb^2+^ on MSBPM; (**b**) pH variation in solution with time during adsorption. (adsorption conditions: No. B in Table 1).

The pH value of the solution is one of the key factors affecting the adsorption performance of the adsorbent. Figure 6a shows the saturated adsorption capacity of two adsorbents for Pb^2+^ at different pH values. As the pH increases, the adsorption capacity of the two adsorbents for Pb^2+^ gradually increases. Pb^2+^ is easily dissolved in acidic solution due to the high concentration of H^+^ in the solution when the pH value of the solution is low, and H^+^ occupies the main adsorption sites of the adsorbent, resulting in a lower adsorption capacity of the adsorbent for Pb^2+^ [25]. Increasing the pH of the solution is beneficial to the adsorption of Pb^2+^ by the adsorbent, but Pb^2+^ will form Pb(OH)_2_ precipitate when the pH of the solution is greater than 7 [26]. In order to avoid the precipitation of Pb^2+^, all subsequent adsorption experiments were conducted in a solution with the pH of 6. The adsorption capacity of MSBPM-Al for Pb^2+^ is slightly higher than that of MSBPM-H_2_O_2_ when the pH is 6.

Figure 6b shows the change in pH with time during the adsorption process of the two adsorbents. The pH of the water solution increased to 5.05 after adding the adsorbent MSBPM-H_2_O_2_ due to its weak alkalinity when the initial concentration of Pb^2+^ was 500 mg·L^−1^ and the initial pH of the solution was 4.7. The pH of the solution slightly increased after adsorption and stabilized at around 5.3 when the solution pH was not adjusted before adsorption. The pH of the solution decreased to 5.7 after adsorption when the solution pH was adjusted to 6 using a 0.5 mol·L^−1^ NaOH solution before adsorption, indicating that the pH of the solution remained below 6 during the adsorption process and Pb^2+^ in the solution did not precipitate. Similarly, the pH of the solution remained below 6 during the adsorption process and Pb^2+^ in the solution did not precipitate when MSBPM-Al was used as the adsorbent.

#### 3.3.3. Effects of Adsorbent Dosage

**Figure 7:**
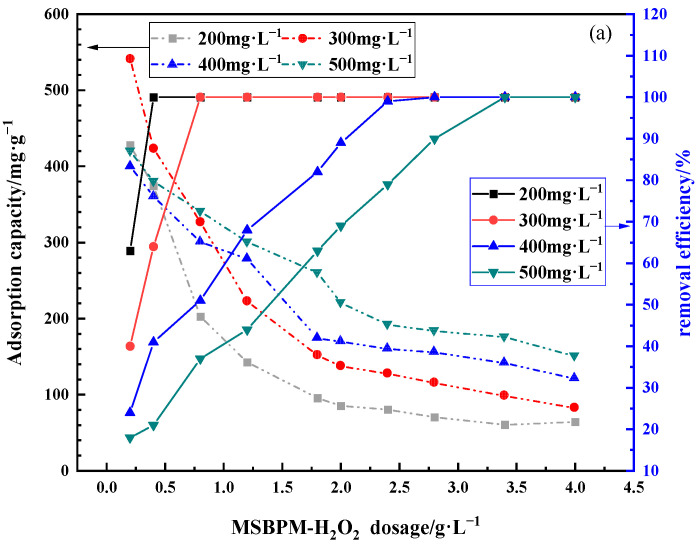

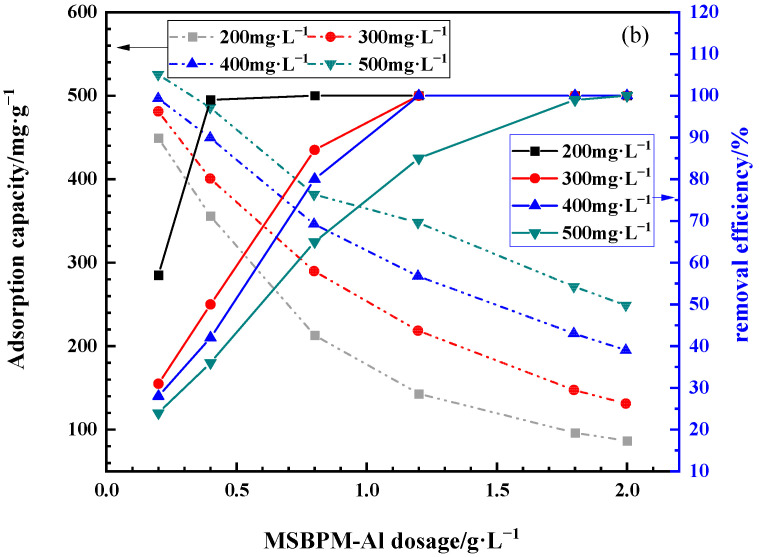
Effect of (**a**) MSBPM-H_2_O_2_ and (**b**) MSBPM-Al dosage on the adsorption capacity and removal efficiency of Pb^2+^ under various initial concentrations (adsorption conditions: No. C in Table 1).

The adsorption capacity and removal efficiency of the adsorbent for Pb^2+^ are closely related to the initial concentration of Pb^2+^ and the dosage of the adsorbent. As shown in Figure 7a,b, as the dosage of the two adsorbents increase, the removal efficiency of Pb^2+^ gradually increased. The removal efficiency of Pb^2+^ can reach 99.99% because the significant increase in the number of adsorption sites in the solution. Only 3.4 g of MSBPM-H_2_O_2_ and 1.8 g of MSBPM-Al per liter of wastewater were required to remove over 99.99% of Pb^2+^ when the initial concentration of Pb^2+^ was 500 mg·L^−1^. The amount of MSBPM-Al required to remove over 99.99% of Pb^2+^ is obviously lower than that of MSBPM-H_2_O_2_, and the adsorption capacity of MSBPM-Al is higher when the initial concentration of Pb^2+^ is higher than 400 mg·L^−1^. However, some of the adsorption sites on the adsorbent cannot be effectively utilized due to the limited amount of Pb^2+^ in the solution with the increase in the dosage of the adsorbent.

#### 3.3.4. Adsorption Mechanism

The adsorption isotherm can be used to describe the interaction between two adsorbent materials and Pb^2+^, and can also serve as a reference for optimizing the performance of MSBPM-H_2_O_2_ and MSBPM-Al. The adsorption performance of the adsorbent can be analyzed by studying the adsorption isotherm of the adsorbent for Pb^2+^. The Langmuir and Freundlich models were commonly used to describe the adsorption process. The Langmuir and Freundlich adsorption isotherm equations are as follows [27]:

(3) $$\frac{C_{e}}{q_{e}}=\frac{1}{q_{m}K_{L}}+\left[\frac{1}{q_{m}}\right]C_{e}$$

(4) $$\log q_{e}=\log K_{F}+\frac{1}{n}\log C_{e}$$

where *C*_e_ is the equilibrium concentration of Pb^2+^ (mg·L^−1^), *q*_e_ is the amount of Pb^2+^ adsorbed at equilibrium (mg·g^−1^), *q*_m_ is the maximum adsorption capacity (mg·g^−1^), and *K*_L_ (L·mg^−1^) is the Langmuir isotherm constant. *K*_F_ (mg·g^−1^) and 1/*n* (dimensionless) are the constants of a Freundlich isotherm.

**Figure 8:**
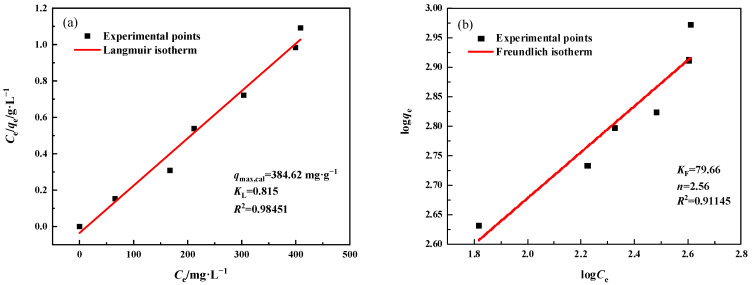
(**a**) Langmuir and (**b**) Freundlich isotherm plots for Pb^2+^ adsorption by MSBPM-H_2_O_2_ (adsorption conditions: No. D in Table 1).

**Figure 9:**
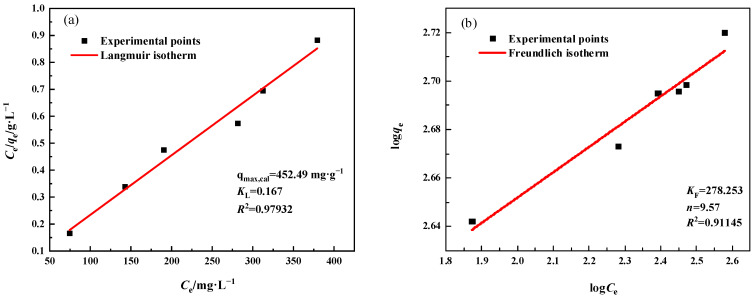
(**a**) Langmuir and (**b**) Freundlich isotherm plots for Pb^2+^ adsorption by MSBPM-Al adsorption conditions: No. D in Table 1).

Figure 8 displayed the Langmuir and Freundlich isotherm models fitted to the adsorption data of the MSBPM-H_2_O_2_ adsorbent material. For the MSBPM-H_2_O_2_ adsorbent material, the correlation coefficient of the Langmuir isotherm model (*R*^2^ = 0.9845) was higher than that of the Freundlich isotherm model (*R*^2^ = 0.9115). Moreover, the maximum adsorption capacity calculated using the Langmuir model was 384.62 mg·g^−1^, which was closer to the experimental value (396.11 mg·g^−1^). Similarly, for the MSBPM-Al adsorbent material, the Langmuir isotherm model fit had a higher correlation coefficient than the Freundlich isotherm model fit (Figure 9), and the maximum adsorption capacity calculated using the Langmuir model was 452.49 mg·g^−1^, which was closer to the experimental value (424.89 mg·g^−1^). These results indicate that the Langmuir model is better suited to describe the adsorption process of the adsorbent for Pb^2+^. Specifically, each Pb^2+^ molecule as an adsorbate is adsorbed onto the surface of MSBPM-H_2_O_2_ with equal adsorption activation energy, and the adsorption process is uniform and belongs to monolayer adsorption [28].

Adsorption kinetics is an important basis for the scientific design of adsorbents and has significant implications for their potential applications. The pseudo-first-order and pseudo-second-order kinetic models were used to investigate the adsorption kinetics of Pb^2+^ by MSBPM-H_2_O_2_ and MSBPM-Al. The equations of these models are expressed by Equations (5) and (6) [29].

(5) $$\log\left(q_{e}-q_{t}\right)=\log q_{e}-\frac{k_{1}}{2.303}t$$

(6) $$\frac{t}{q_{t}}=\frac{1}{k_{2}q_{e}^{2}}+\frac{1}{q_{e}}t$$

where, *q*_e_ and *q*_t_ are the amounts of Pb^2+^ adsorbed per unit of the adsorbent (mg·g^−1^) at equilibrium time and at a given time *t*, respectively; *k*_1_ and *k*_2_ are the rate constant of pseudo-first-order and pseudo-second-order kinetic models, respectively, for adsorption.

**Figure 10:**
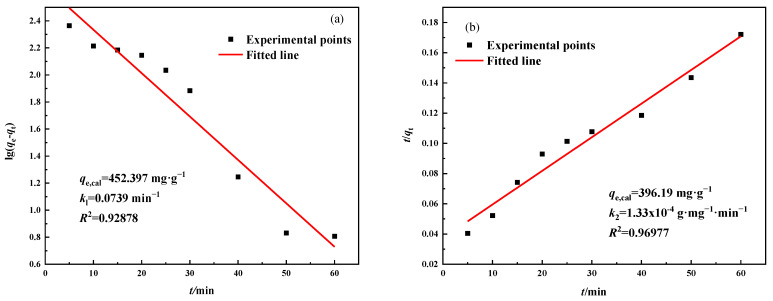
(**a**) Pseudo-first-order and (**b**) Pseudo-second-order model plots for Pb^2+^ adsorption on MSBPM-H_2_O_2_ adsorbent (adsorption conditions: No. E in Table 1).

**Figure 11:**
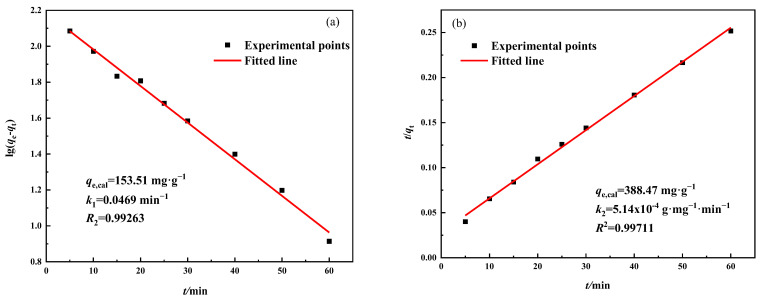
(**a**) Pseudo-first-order and (**b**) Pseudo-second-order model plots for Pb^2+^ adsorption on MSBPM-Al adsorbent (adsorption conditions: No. E in Table 1).

Figure 10 presents the results of the pseudo-first-order and pseudo-second-order kinetic models fitted to the adsorption data of the MSBPM-H_2_O_2_ adsorbent material. Typically, the pseudo-first-order kinetic model better fits the initial adsorption process, where the longer the transfer time of the solute, the lower the concentration of the solution ions at the adsorption equilibrium, and the greater the adsorption capacity. The pseudo-second-order kinetic model more fully describes the diffusion and adsorption process, accompanied by the formation of chemical bonds [30]. For the MSBPM-H_2_O_2_ adsorbent material, the correlation coefficient of the pseudo-second-order kinetic model (*R*^2^ = 0.9698) was higher than that of the pseudo-first-order kinetic model (*R*^2^ = 0.9288), and the calculated maximum adsorption capacity was 396.19 mg·g^−1^, which was closer to the experimental value (396.11 mg·g^−1^). Similarly, for MSBPM-Al adsorbent materials, the correlation coefficient obtained from fitting the pseudo-second-order kinetic model is higher than that obtained from fitting the pseudo-first-order kinetic model (Figure 11). The calculated maximum adsorption capacity is 388.47 mg·g^−1^, which is closer to the experimental value (424.89 mg·g^−1^). The results indicate that the adsorption of Pb^2+^ by both MSBPM-H_2_O_2_ and MSBPM-Al follows the pseudo-second-order kinetic model, and the adsorption process is mainly dominated by chemical adsorption [31,32].

## 4. Conclusions

In this work, the magnesium slag-based porous materials (MSBPM) were successfully synthesized and used as an adsorbent to remove Pb^2+^ from an aqueous solution. The amount of alkali, modulus of water glass, and type and amount of foaming agent had significant effects on the properties of the MSBPM. As the foaming agent dosage increased, the 28-day compressive strength and the apparent density of the MSBPM decreased, while the porosity of the matrix increased. The apparent density and porosity of MSBPM with H_2_O_2_ as a foaming agent ranged from 820 to 1160 kg/m^3^ and from 61% to 48%, respectively. The apparent density and porosity of MSBPM with aluminum powder as a foaming agent ranged from 1610 to 1830 kg/m^3^ and 35% to 23%, respectively. The adsorption capacity of Al powder-foamed MSBPM for Pb^2+^ was generally higher than that of H_2_O_2_-foamed MSBPM. The optimal adsorption capacities of MSBPM-H_2_O_2_ and MSBPM-Al for Pb^2+^ were 396.11 mg·g^−1^ and 424.89 mg·g^−1^, respectively. Only 3.4 g of MSBPM-H_2_O_2_ and 1.8 g of MSBPM-Al per liter of wastewater were required to remove over 99.99% of Pb^2+^ when the initial concentration of Pb^2+^ was 500 mg·L^−1^. The MSBPM showed excellent Pb^2+^ adsorption capacity. The adsorption process of Pb^2+^ on MSBPM-H_2_O_2_ and MSBPM-Al could be accurately described by the Langmuir isotherm model, and the kinetics of both adsorbents followed the pseudo-second-order kinetic model, indicating that the adsorption process was a single-molecule layer chemical adsorption. These results demonstrate that the MSBPM is a low-cost and efficient Pb^2+^ adsorbent.

## Figures and Tables

**Table 1:** Conditions for adsorption experiments.

Series	pH	Initial Pb^2+^ Concentration/mg·L^−1^	Adsorbent Dosage/g·L^−1^	Contact Time/min	Temperature/°C
A	6	500	0.4	120	25
B	3; 4; 5; 6; 7	500	0.4	120	25
C	6	200; 300; 400; 500	0.2; 0.4; 0.8; 1.2; 1.8; 2.0; 2.4; 2.8; 3.4; 4.0	120	25
D	6	200; 300; 350; 400; 450; 500	0.4	120	25
E	6	500	0.4	5; 10; 15; 20; 25; 30; 40; 50; 60	25

**Table 2:** Main chemical composition of magnesium slag.

Sample	Major Composition/% (Mass)					
	CaO	SiO_2_	Fe_2_O_3_	MgO	Al_2_O_3_	Other
Magnesium slag	60.4	30.6	2.57	2.06	1.14	3.32

**Table 3:** Synthesis conditions for MSBPM preparation and 28-days compressive strength.

Foaming Agent	Mixes	Magnesium Slag/g	Water Glass/g	NaOH/g	Adding Water/g	Foaming Agent Dosage/g	Compressive Strength/MPa
H_2_O_2_	1–8%–1.0	150	44.8	10.8	44.35	1.5	2.86
	3–8%–1.0	150	44.8	10.8	42.25	4.5	0.61
	5–8%–1.0	150	44.8	10.8	40.15	7.5	0.15
	8–8%–1.0	150	44.8	10.8	37	12	/
	10–8%–1.0	150	44.8	10.8	34.9	15	0.12
	1–8%–1.3	150	58.3	9.4	35.45	1.5	2.7
	3–8%–1.3	150	58.3	9.4	33.35	4.5	0.49
	5–8%–1.3	150	58.3	9.4	31.25	7.5	0.95
	8–8%–1.3	150	58.3	9.4	28.1	12	0.2
	10–8%–1.3	150	58.3	9.4	26	15	0.16
	1–9%–1.0	150	50.4	12.2	40.65	1.5	2.76
	3–9%–1.0	150	50.4	12.2	38.55	4.5	5.14
	5–9%–1.0	150	50.4	12.2	36.45	7.5	1.88
	8–9%–1.0	150	50.4	12.2	33.3	12	0.36
	10–9%–1.0	150	50.4	12.2	31.2	15	0.22
	1–9%–1.3	150	65.6	10.6	30.65	1.5	7.6
	3–9%–1.3	150	65.6	10.6	28.55	4.5	8.46
	5–9%–1.3	150	65.6	10.6	26.45	7.5	4.7
	8–9%–1.3	150	65.6	10.6	23.3	12	2.2
	10–9%–1.3	150	65.6	10.6	21.2	15	1.8
Aluminum powder	0.5‰–8%–1.0	150	44.8	10.8	45.4	0.075	7.4
	1‰–8%–1.0	150	44.8	10.8	45.4	0.15	6.1
	1.5‰–8%–1.0	150	44.8	10.8	45.4	0.225	5.27
	2‰–8%–1.0	150	44.8	10.8	45.4	0.3	2.1
	0.5‰–8%–1.3	150	58.3	9.4	36.5	0.075	10.7
	1‰–8%–1.3	150	58.3	9.4	36.5	0.15	9.35
	1.5‰–8%–1.3	150	58.3	9.4	36.5	0.225	7.7
	2‰–8%–1.3	150	58.3	9.4	36.5	0.3	3
	0.5‰–9%–1.0	150	50.4	12.2	41.7	0.075	8.4
	1‰–9%–1.0	150	50.4	12.2	41.7	0.15	7.45
	1.5‰–9%–1.0	150	50.4	12.2	41.7	0.225	7.55
	2‰–9%–1.0	150	50.4	12.2	41.7	0.3	6.8
	0.5‰–9%–1.3	150	65.6	10.6	31.7	0.075	12.2
	1‰–9%–1.3	150	65.6	10.6	31.7	0.15	13.5
	1.5‰–9%–1.3	150	65.6	10.6	31.7	0.225	12.1
	2‰–9%–1.3	150	65.6	10.6	31.7	0.3	13.05

**Table 4:** Adsorption capacity of Pb^2+^ by MSBPM. (adsorption conditions: No. A in Table 1).

Foaming Agent	Mixes	Compressive Strength (28 d)/MPa	Adsorption Capacity/mg·g^−1^
H_2_O_2_	1–9%–1.3	7.6	370.02
	3–9%–1.3	8.46	396.11
	5–9%–1.3	4.7	271.72
	8–9%–1.3	2.2	297.68
	10–9%–1.3	1.8	285.27
Aluminum powder	0.5‰–8%–1.0	7.4	339.15
	1‰–8%–1.0	6.1	335.81
	1.5‰–8%–1.0	5.27	424.89
	2‰–8%–1.0	2.1	382.68
	0.5‰–8%–1.3	10.7	355.38
	1‰–8%–1.3	9.35	366.76
	1.5‰–8%–1.3	7.7	272.12
	2‰–8%–1.3	3	456.00
	0.5‰–8%–1.0	7.4	339.15

## Data Availability

The data presented in this study are available on request from the corresponding author.
